# Empirical Distributions of *F*_ST_ from Large-Scale Human Polymorphism Data

**DOI:** 10.1371/journal.pone.0049837

**Published:** 2012-11-21

**Authors:** Eran Elhaik

**Affiliations:** 1 Department of Mental Health, Johns Hopkins University Bloomberg School of Public Health, Baltimore, Maryland, United States of America; 2 McKusick-Nathans Institute of Genetic Medicine, Johns Hopkins University School of Medicine, Baltimore, Maryland, United States of America; Aarhus University, Denmark

## Abstract

Studies of the apportionment of human genetic variation have long established that most human variation is within population groups and that the additional variation between population groups is small but greatest when comparing different continental populations. These studies often used Wright’s *F*_ST_ that apportions the standardized variance in allele frequencies within and between population groups. Because local adaptations increase population differentiation, high-*F*_ST_ may be found at closely linked loci under selection and used to identify genes undergoing directional or heterotic selection. We re-examined these processes using HapMap data. We analyzed 3 million SNPs on 602 samples from eight worldwide populations and a consensus subset of 1 million SNPs found in all populations. We identified four major features of the data: First, a hierarchically *F*_ST_ analysis showed that only a paucity (12%) of the total genetic variation is distributed between continental populations and even a lesser genetic variation (1%) is found between intra-continental populations. Second, the global *F*_ST_ distribution closely follows an exponential distribution. Third, although the overall *F*_ST_ distribution is similarly shaped (inverse J), *F*_ST_ distributions varies markedly by allele frequency when divided into non-overlapping groups by allele frequency range. Because the mean allele frequency is a crude indicator of allele age, these distributions mark the time-dependent change in genetic differentiation. Finally, the change in mean-*F*_ST_ of these groups is linear in allele frequency. These results suggest that investigating the extremes of the *F*_ST_ distribution for each allele frequency group is more efficient for detecting selection. Consequently, we demonstrate that such extreme SNPs are more clustered along the chromosomes than expected from linkage disequilibrium for each allele frequency group. These genomic regions are therefore likely candidates for natural selection.

## Introduction

Knowledge about population genetic structure is central to the study of human origins, DNA forensics, and complex diseases. The present-day genetic diversity observed among human populations was shaped by biological and demographic events that marked their signatures in the genome. Processes such as selection and genetic drift increased the frequency of rare alleles and the genetic diversity among populations [1]. Concurrently, opposing demographical processes, like migration and admixture, reduced the genetic diversity by homogenizing the allele frequencies across populations. Unfortunately, as with most reconstructions, the only recoverable events are those that involved a reasonably large number of individuals and occurred before local migration exchange balanced their effect. Before these genetic signatures can be deciphered and used to unravel the forces responsible for the genetic diversity at each locus, several key questions should be answered: how does geography affect the distribution of genetic information, what is the amount of genetic diversity among human populations, and how does genetic diversity distribute within and between populations?

It is well established that the genetic diversity among human populations is low [2], [3], although the distribution of the genetic diversity was only roughly estimated. Early studies argued that 85–90% of the genetic variation is found within individuals residing in the same populations within continents (intra-continental populations) and only an additional 10–15% is found between populations of different continents (continental populations) [4], [5], [6], [7], [8]. Later studies based on hundreds of thousands single-nucleotide polymorphism (SNPs) suggested that the genetic diversity between continental populations is even smaller and accounts for 3 to 7% [9], [10], [11], [12], [13], [14]. The 1000 Genomes Project’s estimation of the pairwise genetic diversity between continental populations ranged from 5 to 8.3% [3]. Most of these studies have used the *F*_ST_ statistics [15], [16], [17] or closely related statistics [18], [19] and support Lewontin’s [6] findings that humans vary only a little at the DNA level and that only a small percentage of this variation separates continental populations.

However, these interpretations should be treated with caution for several reasons: first, many studies used a small number of polymorphic SNPs (up to 100 SNPs in the nineties and up to 40,000 in the last millennia) – reflecting a limited genetic diversity – or are based on a small number of samples from few populations that do not capture the genetic diversity of the global human population. Second, even for larger datasets (half a million markers) the usefulness for learning about history and natural selection has been limited due to biases in the ways polymorphisms were chosen [20] and their inadequate representation of the underlying true global allele frequency distribution. Third, many studies report the pairwise *F*_ST_ between populations [e.g., 21], an approach that suffers from several caveats [22], and incorrectly estimates the genetic diversity of human populations. Fourth, because finding rare alleles requires large sample sizes, often only common SNPs are studied and rare alleles are absent or under-represented, thus biasing the *F*_ST_ upward. Rare alleles were shown to have a major impact on population structure and must be considered when studying the global genetic diversity [1], [3].

Wright’s *F*-statistics describe the level of heterozygosity in each level of a hierarchically subdivided population [15], [23]. More specifically, *F*-statistics relate the departure from panmixia in the total population and within subpopulations to the total homozygosity due to the Wahlund effect between subpopulations. For a population with a hierarchical structure of three levels – individuals (*I*), subpopulations (*S*), and total population (*T*) – *F*-statistics quantify the genetic differentiation at each level using three indices: *F*_IT_, *F*_IS_, and *F*_ST_ (see supplementary text *F-statistics for measuring population differentiation*). The most commonly reported statistic, *F*_ST_, measures the differentiation of a subpopulation relative to the total population and is directly related to the variance in allele frequency between subpopulations [2]. The mean and variance of *F*_ST_ depend on several factors such as allele frequencies, population subdivisions, and demographic processes and are difficult to be predicted analytically in the absence of the complete genomewide *F*_ST_ distribution [24], [25], [26]. As a result, the mean *F*_ST_ calculated from a subset of the *F*_ST_ distribution is often used to quantify the overall genetic divergence between human populations [e.g., 21].

A widely used approach to detect regions under selective pressure is to compare single-locus *F*_ST_ to the genomewide background *F*_ST_ [e.g., 27,28]. The rational is pan-selectionist; if natural selection favors one allele over others at a particular locus in some populations, the *F*_ST_ at that locus would be larger than *F*_ST_ at other loci in which most differences between populations are due to random genetic drift. However, this approach is not straightforward because extreme population differentiation by itself cannot be assumed to be indicative of a recent population-specific positive selection. In constructing the *F*-statistics model, Wright assumed an infinite number of populations [16], but in practice, the number of populations is often small, and *F*-statistics are strongly subjected to random genetic drift [24]. Moreover, consistently high-*F*_ST_ values over short distances may be due to strong linkage disequilibrium (LD) not selection [2], [29]. Similarly, certain demographic processes can increase the genetic differentiation among populations, for example, by reducing their effective sizes [30], [31], [32], [33]. Although genetic drift and demographic processes affect the entire genome, whereas selection acts on particular genomic regions, distinguishing between *F*_ST_ values driven by each process remains a challenging task that requires a sufficiently large SNP catalog. Such a comprehensive SNP catalog became recently available in the third HapMap phase [34]. The HapMap project endeavored to map the majority of common and rare variants throughout the genome and provide a large and dense SNP map. HapMap thus enables us to calculate the population differentiation more accurately using individuals with ancestry from different parts of Africa, Europe, and Asia.

**Figure 1:**
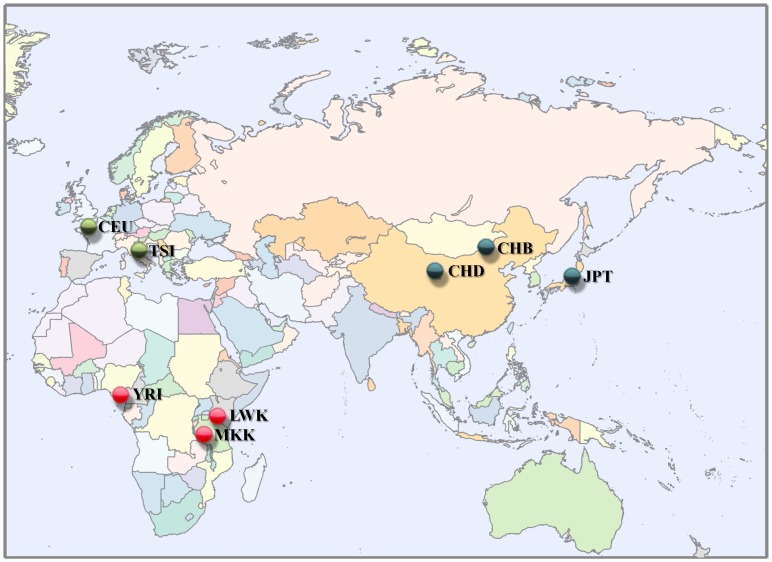
Map of the Old World. The geographical regions of origins are shown for the eight populations used in this study. Intra-continental populations have the same color.

Here, we study the extent of genetic differentiation in eight human populations ascribed to three continental populations and their intra-continental populations (Figure 1). We estimate the global genetic diversity in a hierarchical manner over 1 million markers. To the best of our knowledge, this is the most extensive effort to describe the genetic diversity distribution in humans. We further address long standing questions of the shape of the *F*_ST_ distribution, its mean, and its variance [22], [24], [25], [35], [36], [37], which are critical in population genetic studies [25]. We compare the shape of the overall *F*_ST_ distribution to that obtained from SNPs grouped by minor allele frequency (in 0.1 increments from 0 to 0.5 minor allele frequency) and derive a linear equation to describe the relationship between *F*_ST_ and the mean minor allele frequency. We also compare the clustering of high-*F*_ST_ SNPs along chromosomes in each allele frequency group to the clustering expected from linkage disequilibrium. Last, we devise a strategy to detect genomic regions candidate for natural selection.

## Results

### Data Description

HapMap phase 3 (second draft) includes new populations and additional samples to existing populations genotyped in previous phases [34]. Over 1 million SNPs were added to the new and existing populations (Figure S1 and Table S1). The number of HapMap phase 3 SNPs and individuals that passed our quality control criteria (“QC++,” see Text S1 *Assessing Data Quality*) and used for subsequent analyses is shown in Table S1. The QC++ data for 602 samples was used to construct a “continental” dataset with ∼3 million SNPs that were genotyped in at least one population of each continent and a smaller “intra-continental” dataset with ∼1 million SNPs that were genotyped in all eight populations.

**Figure 2:**
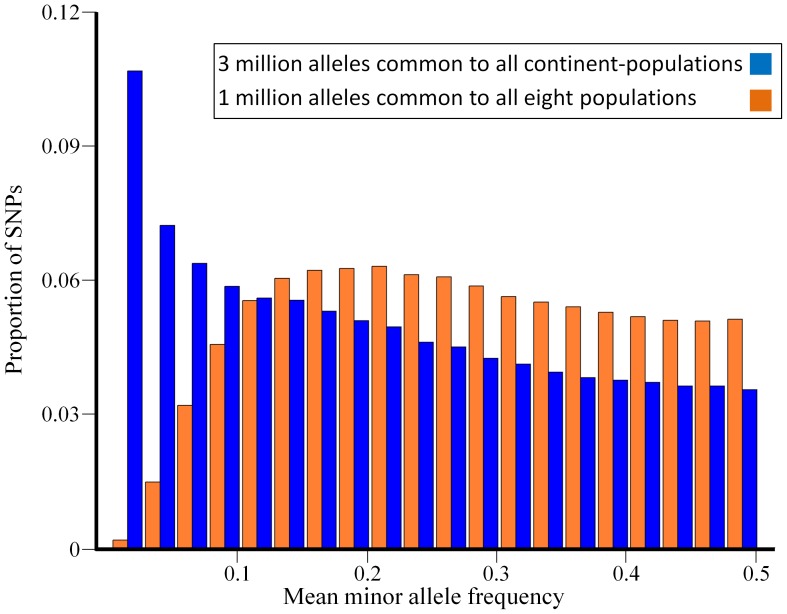
Minor allele frequency distributions for autosomal SNPs.

In the continental dataset, over 82% of the SNPs are common (minor allele frequency (MAF) ≥0.05) and less than 5% are considered rare (MAF <0.01). A comparison of the MAF distributions between the continental and the intra-continental datasets reveals gross differences in allele frequencies (Figure 2): for example, the continental dataset consists of three orders of magnitude more rare SNPs than the intra-continental dataset. The reason for these differences is the large number of rare ENCODE SNPs genotyped only in the four original HapMap populations and thus were excluded from the intra-continental dataset (Figure S1 and Table S1).

### Inferring the Genetic Variation in a Hierarchical Population Structure Using

**Figure 3:**
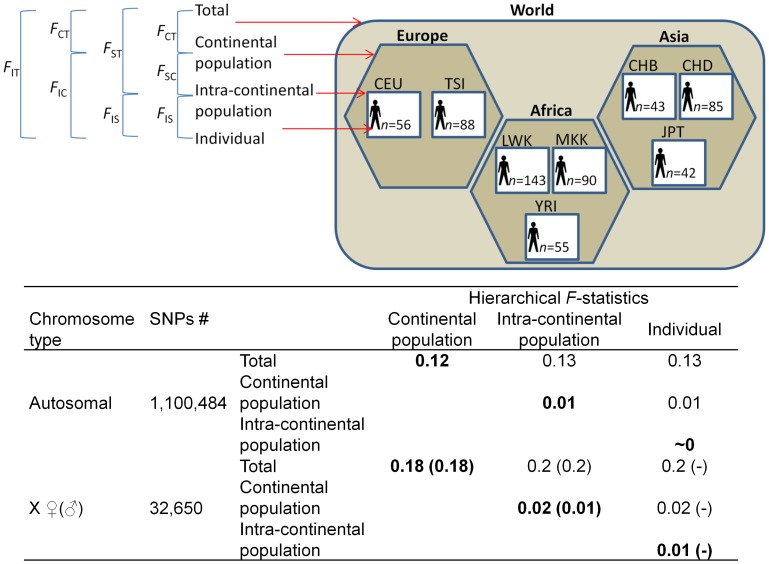
An illustration of a hierarchical *F*-statistics analysis using eight populations. Samples are organized in a three level structure of individuals, intra-continental populations, and continental populations. The relationships between the six fixation indices are depicted on the top left and follow the formulation of Eq. S1. For example, 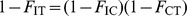. Below are the *F*-statistics, calculated separately for autosomes, male X-chromosomes, and females X-chromosomes. The indices measuring the genetic variation between continental populations (*F*_CT_), between intra-continental populations (*F*_SC_), and between individuals of intra-continental populations (*F*_IS_) are shown in bold.

Looking at the intra-continental population dataset, worldwide human populations can be divided into the three Old World continental populations and further subdivided to intra-continental populations and finally individuals. The components of variance for a population structure with three hierarchical levels were obtained using *F*-statistics (Figure 3). The key *F*-statistics 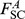 and 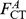 describe the variation in autosomes ascribed to intra-continental variation nested within each continent and geographical separation between continents, respectively. The vast majority of genetic variation in autosomes (1−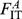 = 87%) is found within individuals. Only a paucity of the total genetic variation (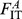 = 13%) is distributed between continental populations (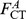 = 12%) and an even lesser amount (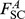 = 1%) between intra-continental populations. As expected from their dosage in the population, *F*-statistics were slightly higher in the X chromosome than in autosomes with most genetic variation (1−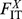 = 80%) found within individuals, whereas the large portion of the total genetic variation (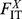 = 20%) is distributed between continental populations (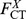 = 18%). Only a small variation amount (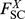 = 2%) is distributed between intra-continental populations (Figure 3). Similar results were obtained for males and females. Individuals in intra-continental populations are under panmixia (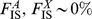) and their allele frequencies do not deviate from the Hardy-Weinberg equilibrium. To test the affect of rare alleles on the genetic variation, we excluded rare alleles (MAF ≤0.05) and repeated the analysis. The results did not change.

### Calculating the Empirical Genomewide Distribution of *F*_ST_

**Figure 4:**
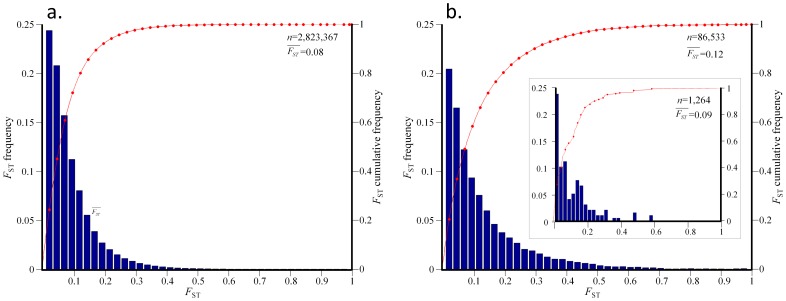
Distribution of locus-specific *F*_ST_ in three continental populations (CEU+TSI, CHB+CHD+JPT, LWK+MKK+YRI). *F*_ST_ values were obtained for (a) 2,823,367 autosomal SNPs and (b) 86,533 SNPs on the non-recombining region of the X chromosome and 1,264 SNPs on the PAR region (inset). The histograms show bin distribution as indicated on the x-axis and the cumulative distribution (line).

Because the major portion of genetic variation is distributed between continental populations (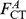 = 12%) we used the continental dataset to further investigate the behavior of the *F*_ST_ (i.e., 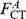) distribution. Compared to the 1 million SNPs of the intra-continental population dataset, the continental dataset contains additional two million SNPs, many of which are rare, that reduce the mean *F*_ST_ compared to that reported herein. The empirical *F*_ST_ distribution was plotted for autosomes and for the recombining and nonrecombining (PAR) regions of the X chromosome (Figure 4).

For autosomal SNPs, the *F*_ST_ distribution is right-skewed with a mean and standard deviation of 0.08 (Figure 4a). The biological interpretation of these values is a moderate genetic differentiation [17]. The *F*_ST_ distribution is a thin-tailed distribution (0.7% of SNPs have *F*_ST_ ≥0.4) that sharply declines. These results are contrary to previous descriptions of a slowly declining *F*_ST_ distribution with high SNP densities at the tail; for example, Akey et al. [35] calculated an *F*_ST_ distribution (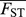 = 0.12), in which 6% of the SNPs had *F*_ST_ ≥0.4 using 25,549 autosomal SNPs genotyped in African-American, East Asian, and European-American. A later study [38] analyzed 8,525 autosomal SNPs in 84 African-American, European-American, Chinese, and Japanese individuals described an *F*_ST_ distribution (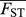 = 0.13) with a thinner tail (4% of SNPs have *F*_ST_ ≥0.4). These reports relied on relatively small samples of common SNPs from admixed populations that do not represent the worldwide distribution of genetic variation.

The *F*_ST_ distribution closely follows an exponential distribution with *λ* = 12.5, even though the Kolmogorov-Smirnov test rejected that hypothesis (Figure S2). When plotted on a QQ-plot the *F*_ST_ distribution of autosomes is under-dispersed as compared with the expected exponential distribution (Figure S3a). However, when excluding the rarest minor alleles (MAF <0.05) the two distributions approximately fit the line *y* = *x* (Figure S3a). Similar results were obtained for the X-chromosomal *F*_ST_ distribution (Figure S3b), indicating that the skewness in the original *F*_ST_ dataset is caused by the excess of rare alleles. Despite of the large variation in SNP density ranging from 0.7 (chromosome 19) to 1.17 (chromosome 6) SNPs every 1,000 nucleotides, the distributions of *F*_ST_ and MAF have a similar mean and standard deviation for all autosomes (Table S2), suggesting that even chromosomes with poor SNP density allow a good estimation of population genetic statistics.

As expected, the *F*_ST_ distribution for the X-chromosomal PAR region (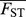 = 0.09) (Figure 3b) is more similar to the autosomal *F*_ST_ distribution (Figure 3a) than the X-chromosomal *F*_ST_ distribution (Figure 3b) in shape and density for both the least diverged SNPs (43% of the SNPs have *F*_ST_ <0.05) and the highly diverged SNPs (0.6% of SNPs have *F*_ST_ ≥0.4). The *F*_ST_ distribution for the X-chromosome (Figure 3b) is also positively-skewed (*γ* = 1.7) and enriched in highly diverged SNPs (5% of SNPs have *F*_ST_ ≥0.4). The distribution follows a near-exponential distribution (*λ* = 8.15) with a moderate decline, compared to the autosomal *F*_ST_ distribution.

The mean X-chromosomal *F*_ST_ distribution is substantially higher than that of autosomal SNPs, consistent with the smaller effective population size or selection on X-linked loci [2]. Assuming a 1∶1 sex ratio, there are four copies of each autosome for every three copies of X chromosome. Therefore, X-linked loci experience a stronger impact of genetic drift that increases their genetic differentiation in a ratio of 3∶4 compared with autosomal loci. We used the *Q* statistic to calculate the *F*_ST_ ratio of autosomes to X chromosomes (Eq. 2) and tested for deviations from an expected *Q* of 0.75 (Eq. 3). We found a significantly lower genetic differentiation between continental populations of *Q* = 0.63±0.01 (bootstrap test, *p*<0.001), indicating that these populations exhibit a smaller genetic differentiation in their X chromosome than expected by chance. This low ratio could be the result of long-range male-migration from Africa that was maintained due to continuous expansions through the time period of when non-African populations formed. Alternative explanations can be a stronger selection on X-linked loci or an accelerated genetic drift assumed to occur in non-African history after the split from Africans.

Wright’s theory of the evolutionary change of *F*-statistics depends on the assumption of infinite number of subpopulations [16], [23]. Because in reality the number of subpopulations is small, many studies relaxed the infinite population size assumption to predict the evolutionary change of *F*_ST_ in a subdivided population of finite size [24], [36]. For example, it has been shown [22], [24], [39] that under neutrality when the number of populations is small (less than four) and the effective population size is small, allele frequencies are strongly susceptible to genetic drift and have an inverse J-shaped *F*_ST_ distribution, whereas for ten or more populations the *F*_ST_ distribution resembles bell-shape. The reason for the inverse J-shaped distribution for fewer populations is due to the high likelihood that all populations will have similar allele frequencies and that in the later generations the same alleles may be fixed in all subpopulations. By contrast, a bell-shaped distribution appears because the chance of the same allele being fixed in many subpopulations is extremely small [24]. Here, we analyzed two datasets, continental and intra-continental, consisting of a small and large number of subpopulations (three and eight, respectively). These datasets share the same effective population size, estimated to be *N_e_* = 10,000 [40], and consist of a large number of SNPs (3 M and 1 M, respectively). These datasets were therefore expected to exhibit an inverse J-shaped and bell-shaped *F*_ST_ distributions, respectively, but instead, both datasets exhibit a similar inverse J-shaped *F*_ST_ distribution (Figures 4, S4). These results reflect the lack of genetic differentiation, in the case of the intra-continental dataset. In other words, although we compared a large number of populations (eight), due to their high genetic similarity, they appear as three populations [41], in agreement with our results from the hierarchical analysis (Figure 3).

### Obtaining *F*_ST_ Distribution for Allele Frequency Groups

**Figure 5:**
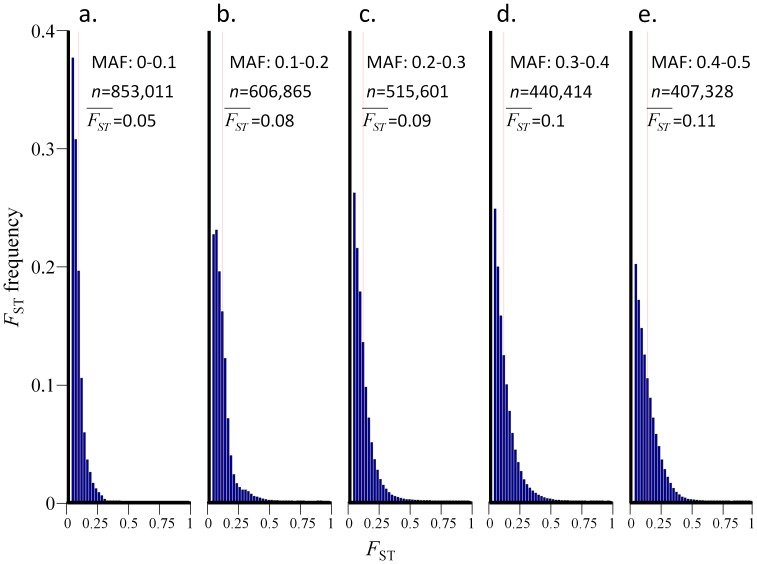
*F*_ST_ distributions for five MAF groups (a–e). The histograms show the *F*_ST_ values for five allele frequency groups divided by their MAF.

Because nearly all the 3 million SNPs in our continental dataset are non-coding, it is reasonable to assume neutrality. Under neutrality, newly introduced variants require a long time to reach high frequencies. During this time, recombination will tend to break down the linkage disequilibrium (LD) between neighboring variants. Consequently, common variants tend to be older [42], [43] and harbored within regions of limited LD [44], [45]. The genomewide *F*_ST_ distribution (Figure 4a) thus includes SNPs with dissimilar allele frequencies and biological properties owing both to the stochastic nature of genetic drift and to the biological importance of the genomic region involved in the process. An *F*_ST_ distribution plotted for SNPs with particular minor allele frequency (Figure 5) is therefore expected to have a unique shape and variance because it describes regions that were likely affected by similar evolutionary forces. Indeed, dividing the SNPs of the continental dataset into five non-overlapping allele frequency groups according to their MAF –0–0.1 (*n* = 853 K), 0.1–0.2 (*n* = 607 K), 0.2–0.3 (*n* = 516 K), 0.3–0.4 (*n* = 440 K), and 0.4–0.5 (*n* = 407 K) – shows distinct shapes for each distribution. The majority of the SNPs (52%) were concentrated in the low-frequency allele groups (0–0.2), whereas only 14% of the SNPs were ascribed to the most common allele frequency group (0.4–0.5). Each *F*_ST_ distribution appears to follow an exponential distribution, even though the Kolmogorov-Smirnov test rejected that hypothesis.

**Figure 6:**
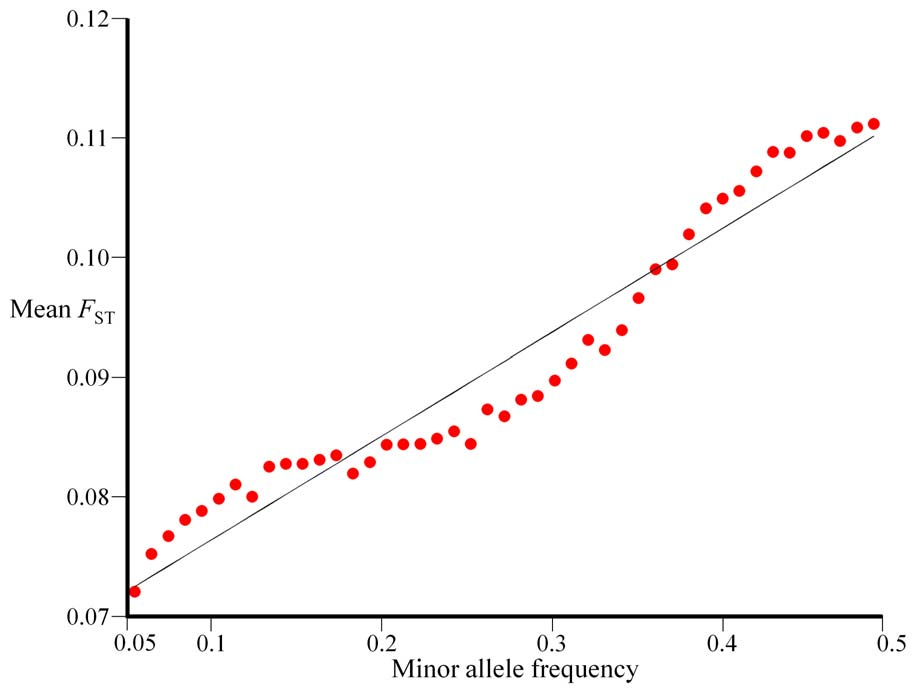
Correlating MAF with *F*_ST_. The mean *F*_ST_ plotted for all MAF groups (dots), excluding the rarest ones (MAF >0.05), allows us to express the correlation between the two variables using a single linear equation (Eq. 4).

To study the relationships between 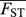 and the MAF, we defined 45 MAF groups, each with a consecutive range (0.05–0.06, 0.06–0.07…0.049–0.5) and divided the SNPs of the continental dataset into these groups based on the MAF of each SNP. Low MAF groups (MAF <0.05) were ignored due to their skewed distribution (Figure 4). Because the *F*_ST_ distribution of each MAF group is very narrow, we used its mean values to study the relationship with the mean MAF. We found a linear relationship between 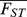 and *MAF* (Figure 6):
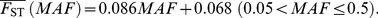
(4)


### Measuring the Dispersal of High-*F*_ST_ SNPs

**Figure 7:**
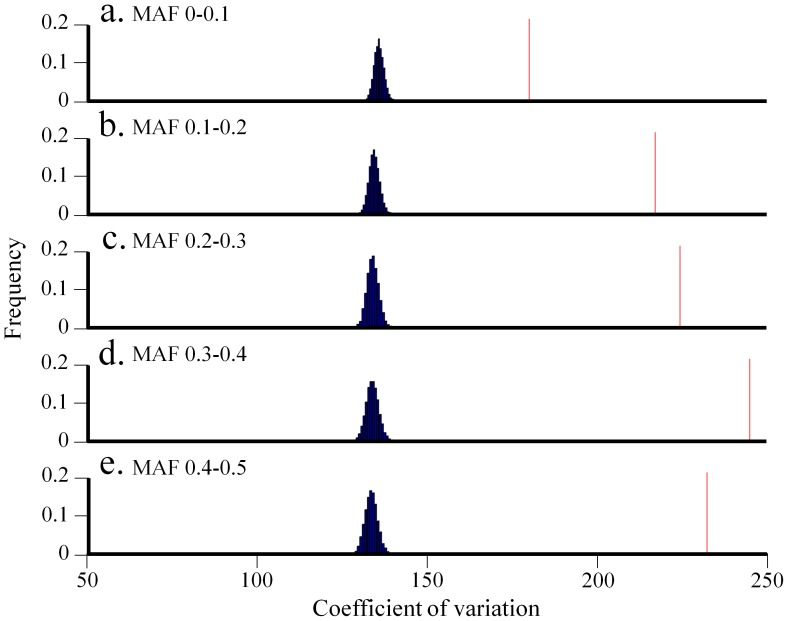
Comparing the coefficient of variation for high- and low-*F*_ST_ SNPs. Frequency distribution of coefficient of variation calculated between adjacent *F*_ST>threshold_ SNPs (line) and between random samples of *F*_ST<threshold_ SNPs (histogram) for five allele frequency groups (a–e).

Because adjacent high-*F*_ST_ SNPs within each allele frequency group are likely to share similar evolutionary history, we hypothesized that they would be more clustered along chromosomes than other SNPs. To test that hypothesis, we picked SNPs with extreme high-*F*_ST_ values from the top 0.005 percentile of each *F*_ST_ distribution (Figure 5). These SNPs were termed “*F*_ST>threshold_,” and all other SNPs “*F*_ST<threshold_.” We compared the coefficient of variation for adjacent *F*_ST>threshold_ and random *F*_ST<threshold_ SNPs and found that *F*_ST>threshold_ SNPs are significantly more clustered for all allele frequency groups (bootstrap test *p*<0.0001) (Figure 7). Similar results were obtained using two other measures of dispersion (quartile coefficient of dispersion and geometric coefficient of variation) and are not shown.

The extent within *F*_ST>threshold_ SNPs clustering along chromosomes is demonstrated in Figure S5. *F*_ST>threshold_ SNPs reside in a very close proximities: 29–42% of the *F*_ST>threshold_ SNP pairs from all allele frequency groups are located within less than 10 kilobases (kb) from each other and 17–25% of them are within 10 kb to 100 kb from each other. Although *F*_ST>threshold_ SNPs from the common allele frequency group (0.4–0.5) accounted for a small fraction of *F*_ST>threshold_ SNPs (14%), the short distances between adjacent SNP pairs suggest high clumping as well.

### Correlating LD between Adjacent SNPs

**Figure 8:**
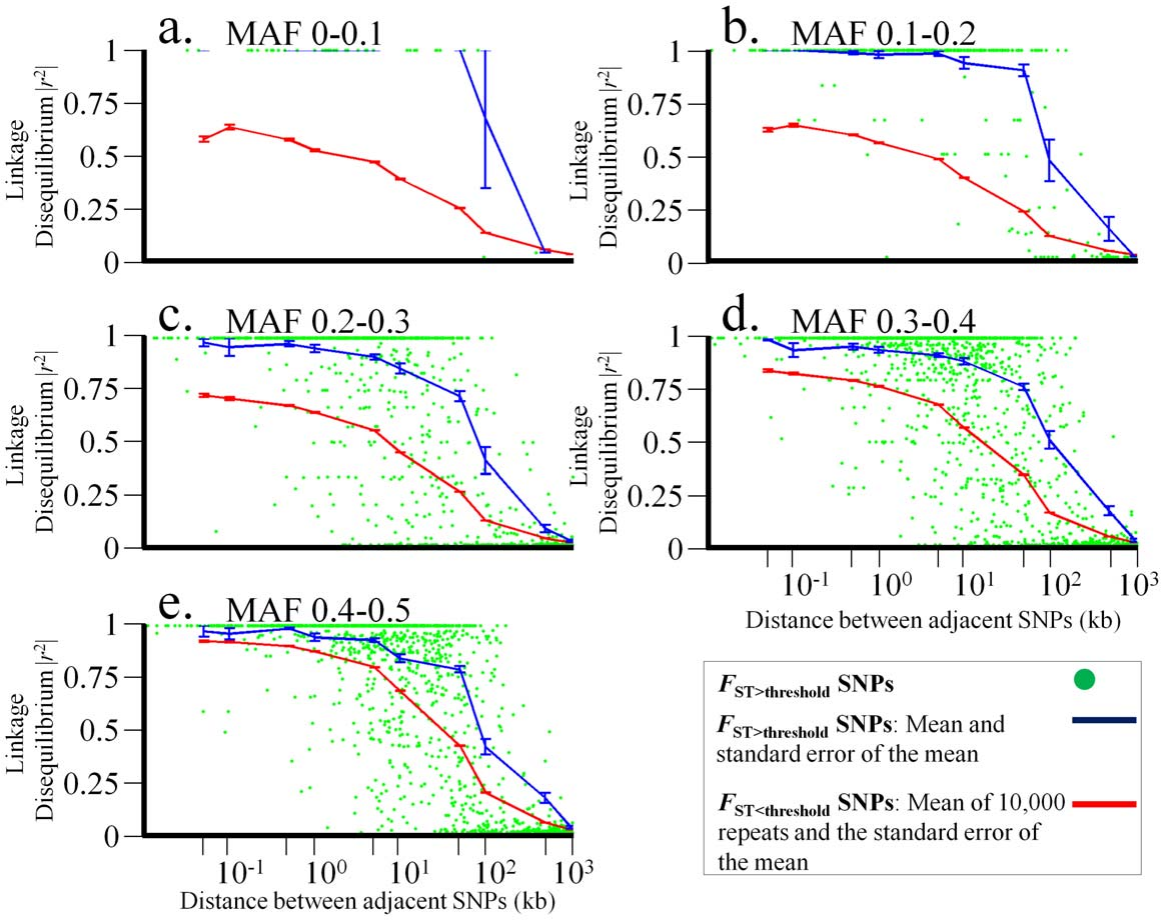
LD for five allele frequency groups as a function of physical distance in Africans. LD (*r^2^*) in African populations is plotted as a function of physical distance on a log-scale for five allele frequency groups (a–e). To simplify the presentation, the mean and standard error of the mean *r^2^* for the *F*_ST >threshold_ SNPs (blue) and *F*_ST<threshold_ (red) are presented for different between-SNP distances (50 bp, 100 bp, 1 kb, 5 kb, 10 kb, 50 kb, 100 kb, 500 kb, and 1000 kb). *F*_ST>threshold_ SNPs are marked as green dots.

The observed clusters of *F*_ST>threshold_ SNPs could have been formed by either the hitchhiking effect of SNPs surrounding a region under natural selection or genetic drift. To test which of these forces shaped the observed clusters, we calculated the LD between adjacent *F*_ST>threshold_ and *F*_ST<threshold_ SNPs for Africans, Europeans, and Asians (Figures 8, S6–S7). We found that the LD (measured as pairwise *r* ^2^) between adjacent *F*_ST>threshold_ SNPs is biphasic: initially high (0–10 kb) and later decays. As expected, we found low LD (*r* ^2^<0.3) when the inter-SNP distances were larger than 100 kb. Non-African populations exhibited a slower decay than African populations over all physical distances. The decay is moderate for common alleles and sharper for low-frequency allele groups.

We found that all *F*_ST>threshold_ SNPs exhibit significantly higher *r* ^2^ values (bootstrap test *p*<0.0001) than *F*_ST<threshold_ up to distance of 1 Mb (Figure 8). The LD for common allele frequency groups (Figure 8d–e) was low over short distances (1–100 kb) and declined slow over large distances (100 kb-1 Mb) compared with the LD for rare allele frequency groups (Figure 8a–b).

Unfortunately, the observed *F*_ST_ and LD patterns can be explained in more than one way. The high-*F*_ST_ in the *F*_ST>threshold_ SNPs indicates large genetic differentiation between populations but their high-LD indicates correlated genetic differentiation. Such genetic differentiation may be the product of selection but can also occur at random by genetic drift. Therefore, the question whether clustered *F*_ST<threshold_ SNPs with high-LD are due to the hitchhiking effect following selection or genetic drift remains to be further tested.

The decline in LD was similar between *F*_ST>threshold_ SNPs (Figures 8d–e) regardless of their allele frequency group, in agreement with [46]. Interestingly, *F*_ST<threshold_ SNPs belonging to different allele frequency groups exhibit disparity in the average decline in LD (100 kb-1 Mb). This disparity can be explained by the clustering of *F*_ST<threshold_ SNPs in LD blocks of different sizes. Eberle et al. [46] showed that low-frequency SNPs (Figures 8a–b) are found in longer LD blocks that often overlap, whereas high-frequency SNPs (Figures 8d–e) are found in much shorter LD blocks that do not overlap. Because of the overlap in long LD blocks, the low-frequency SNPs may appear closer to alleles from other low-MAF groups, but not necessarily SNPs from their MAF group. By contrast, high-frequency SNPs reside in the same short blocks are more likely to be closer to SNPs of their MAF group.

In addition to selection and genetic drift, the overall LD of *F*_ST<threshold_ SNPs was also affected by demographic processes. The variability in the extent of LD between continental populations clearly marks their population history. Africans have the shortest LD (Figure 8), whereas Europeans and Asians have the longest LD (Figures S6–S7). The findings of high-LD for non-African populations are in agreement with models proposing a founding event during the expansion from Africa [47], [48] with a bottleneck that occurred during this period [49], [50]. Therefore, by correcting for the effect of LD we can potentially distinguish selection from other biological and demographic processes acting on *F*_ST>threshold_ SNPs.

## Discussion

Even in the pre-Darwinian era it was clear that human populations vary and that this variation played a critical role in the individual’s development and its phenotypic attributes. The variation between individuals defined the space in which population groups were identified and to which individuals were classified. The post-Darwinian perception was that variation between individuals is the outcome of evolutionary processes that act differently on different individuals, but the extent of the genetic differentiation remained under debate [2], [6], [35], [51].

The comprehensive high-quality HapMap (phase 3, second draft) SNP catalog genotyped over eight worldwide populations is the best approximation to the global genetic diversity available. We therefore used the HapMap catalog to quantify the amount of genetic diversity between and within eight human populations more accurately than previously done [e.g., 4,6,8]. The genetic variation in the population structure was measured using hierarchical *F-*statistics. We showed that individuals of intra-continental populations are under panmixia (Figure 3) and that their allele frequencies do not deviate from the Hardy-Weinberg equilibrium. We further showed that only 12% of the total genetic variation is distributed between continental populations with a minor amount of 1% between intra-continental populations. To illustrate these results, consider an African nomadic tribe that populates a new continent. The new population would preserve 87% of the worldwide human genetic variation. We note that the estimations of genetic variation distributed between continental and intra-continental populations are likely biased upward because, as shown in Figure 4 and elsewhere [3], they do not account for the extensive amount of rare variants. However, it is possible that the small number of populations studied here under-represented the global genetic variation and thus biased the genetic variation downward. Future studies carried on additional populations are necessary to test whether our conclusions hold for worldwide populations.

Our findings suggest that the high migratory rates within continents and the relative ineffectiveness of geographical and socio-economical barriers maintained our shared genetic history and prevented the genetic isolation of the studied populations [5], [52]. The most meaningful barriers to gene flow are the geographical barriers between continents, due to the partial isolation of human populations during a long time throughout their history. The affect of such barriers on the LD is reflected in our findings (Figures 8, S6, and S7).

Many attempts were made to estimate the distribution parameters of *F*_ST_ [24], [25]; however, due to the absence of a comprehensive SNP catalog, the distribution type remained elusive. We first showed that the *F*_ST_ distribution is approximately exponentially distributed (Figure S2) and, consequently, that the distribution shape and variance depend on its mean. Second, we demonstrated that *F*_ST_ distributions vary for different minor allele frequency groups (Figure 5), though they are similar in shape to the genome-wide *F*_ST_ distribution (Figure 4). Third, we found that the change in the mean *F*_ST_ is linearly related to the MAF.

The first results are not surprising. According to Eq. S2, *F*_ST_ depends on the effective population size (*N_e_*) and generation time (*t*), not on the minor allele frequency range. Thus *F*_ST_ is expected to exhibit a similarly-shaped distribution for different minor allele frequency groups. The variation in *F*_ST_ distributions for different MAF groups is explained by the neutral theory. Under neutrality, most of the evolutionary changes are the result of genetic drift acting on neutral alleles, thus the time until a mutation event can be modeled as a Poisson process. This process if memoryless; that is, if an allele did not mutate in time period *t_0_*_,_ it has the same probability to mutate in time period *t_1_* as it had in time period *t_0_*. As expected, this probability is higher for common alleles and lower for rare alleles. We have shown that the measure of genetic differentiation, *F*_ST_, is a random variable that approximately follows an exponential distribution with a mean *λ* (Figure 4). When *F*_ST_ is calculated for *n* allele frequency groups (*f)* it behaves as a random exponential variable with a mean and standard deviation *λ_f_*. Because common alleles are more likely to mutate in any time period than are rare allele, they will exhibit higher *λ_f_* than rare allele in a linear relationship (Figure 6).

Although both genetic drift and selection increase the population differentiation as measured by *F*_ST_, genetic drift randomly alters the allele frequencies among different populations, whereas selection has a very local effect resulting in increased *F*_ST_ in a certain loci due to the hitchhiking effect. Therefore, SNPs with similar minor allele frequencies and high-*F*_ST_ may be targeted when searching for SNPs under natural selection. Identifying the shape of the *F*_ST_ distribution is thus critical to finding SNPs under selection. Because SNPs with similar MAF may share a common origin and demographic history, comparing the *F*_ST_ of SNPs within their MAF group is more informative than comparing them with SNPs from random allele groups.

In the process of LD, variants in physical proximity along a chromosome tend to be more correlated in the population than would be expected at random formation of haplotypes.

The clumping of such variants, unrelated with selection, may also yield high-*F*_ST_ SNPs. Therefore, employing high-*F*_ST_ values to infer population-specific positive selection requires accommodating for the LD effect. Because the age of variants is related to the extent of LD around them [45], it is necessary to group SNPs accordingly to interpret the LD patterns. Under neutral evolution, new variants require a long time to reach high frequencies in the population. Consequently, due to the effect of recombination, the LD around variants will decay substantially over time. Therefore, alleles from the common allele group (0.4–0.5) will typically be older and their LD would be short-ranged, whereas rare alleles that may be either very young or very old will exhibit long- or short-range LD, respectively (Figures 8, S6, and S7).

We note that although this general pattern holds for long intervals, distance by itself does not have a crucial influence on short-range LD. Reduction in LD over short distances due to recombination is low compared with the effects of genetic drift and migration. Moreover, demographic processes, such as founding effect, may produce high-LD over intermediate-range, although these processes are expected to have a smaller effect on African populations (Figure 8). For long-range distances, the recombination frequency would increase and weaken any association caused by biological processes other then strong selection. Therefore, unlike alleles under genetic drift, alleles under natural selection will exhibit high-LD over large distances, relative to their frequency. The genomic regions harboring those SNPs would be likely candidates for natural selection.

Detecting signatures of natural selection and deciphering their causes can shed light on the evolution of the human genome and have practical implication for the search of loci involved in complex disorders. A further study is necessary to identify the clusters of SNPs with high-*F*_ST_ and associate them with genes related to diseases.

## Materials and Methods

### HapMap 3 Genotype Data

The genotype data of individuals from eight relatively homogeneous populations were downloaded from the International HapMap Project web site (phase 3, second draft) at http://hapmap.ncbi.nlm.nih.gov/downloads/genotypes/2009-02_phaseIIIII/forward/non-redundant/ [34]. The eight populations comprised of Utah residents of Northern and Western European ancestry from the CEPH collection (CEU); Han Chinese from Beijing, China (CHB); Chinese from metropolitan Denver, Colorado (CHD); Japanese from Tokyo, Japan (JPT); Luhya in Webuye, Kenya (LWK); Maasai in Kinyawa, Kenya (MKK); Yoruba in Ibadan, Nigeria (YRI); and Italians from Tuscany, Italy (TSI). Three population samples (CEU, MKK, and YRI) are parent-offspring trio populations, and the rest are unrelated individuals. We used only QC+ data from the “non-redundant filtered” dataset. Because we used HapMap 3 draft data, we applied additional data quality filters (see Text S1 *Assessing Data Quality*). SNPs and samples that passed our filtering criteria were termed “QC++” (Table S1).

Because many SNPs were not genotyped in all eight populations, we created two datasets: “continental” with ∼3 million SNPs that were genotyped in at least one population of each continent and “intra-continental” a common subset of ∼1 million SNPs that were genotyped in all eight populations. Y-linked and mitochondrial SNPs were not included in the study due to their small number. Analyses were carried out on the continental dataset, unless stated otherwise.

### Analysis of Hierarchical Population Structure

To study the distribution of genetic diversity between distinct populations, we considered a hierarchical population structure of three levels: individuals (I), intra-continental populations (S), and continental populations (C) (Figure 3). Using the intra-continental dataset, the hierarchical structure was obtained by aggregating 602 individuals (first level), classified to eight intra-continental populations (second level) within three continental populations (third level). Depicting this hierarchical framework with *F*-statistics required six indices: *F*_IS_ that measures the correlation between alleles of individuals relative to the intra-continental population, *F*_SC_ that measures the correlation between alleles of intra-continental population relative to the continental population, and *F*_CT_ that measures the correlation between alleles of continental population relative to the total population. The remaining indices – *F*_IC_, *F*_IT_, and *F*_ST_ – were similarly defined (see also Text S1 *F-statistics for measuring population differentiation*). Hierarchical *F*-statistics were calculated for all autosomal SNPs and separately for males and females X-chromosomal SNPs from the non-recombining regions. The significance of the variation between regions within continents was tested by bootstrap analysis of randomizing individuals between regions of the same continent and repeating the process 10,000 times. Hierarchical *F*-statistics were calculated with the HierFstat package version 0.04–4 [53] that we optimized for large dataset analysis.

### Calculating *F*_ST_

We followed Wright’s [15] method to calculate *F*_ST_. For each SNP, we calculated the frequencies of both alleles in each population. We then identified the allele with the smallest global frequency (*P*) when calculated as a weighted average over all populations so that (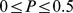). Similarly, the variance of the minor allele frequency 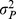 was obtained and *F*_ST_ was calculated as:
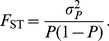
(1)


Although the dynamics of *F*_ST_ were extensively studied, no single model to describe the *F*_ST_ distribution has been proposed [25], [37]. We hypothesized that the *F*_ST_ distribution follows an exponential distribution. To test that hypothesis we used the Kolmogorov-Smirnov test (*α* = 0.01) for a distribution with unknown mean [54].

Comparing estimates of *F*_ST_ for autosomes and X chromosome provides further insights into the demographic history of populations. If the difference *Q* between *F*_ST_ values of autosomes and X chromosome [50], [55] can be derived as:
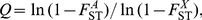
(2)


*Q* is approximately:
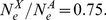
(3)


Deviation from this expectation may indicate different demographic histories for autosomes and X-linked SNPs. The significance of *Q* was estimated by a bootstrap analysis preformed with 10,000 selecting random datasets of 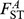 and 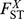 of size 10,000 and using their mean *F*_ST_ values to calculate *Q*.

### Estimation of Data Dispersal

To study the effect of minor allele frequency (MAF) on the shape of the *F*_ST_ distribution, SNPs were divided into five allele frequency groups according to their MAF (0–0.1, 0.1–0.2, 0.2–0.3, 0.3–0.4, and 0.4–0.5). The *F*_ST_ distribution was then calculated for each allele frequency group. The Kolmogorov-Smirnov test (*α* = 0.01) for a distribution with unknown mean [54] was used to test whether each *F*_ST_ distribution follows an exponential distribution.

To study the difference between SNPs with high- and low-*F*_ST_ values, the top 0.005 percentile of each *F*_ST_ distribution was set as a threshold. SNPs with *F*_ST_ values above the threshold were considered *F*_ST>threshold_ SNPs and all other SNPs were considered *F*_ST<threshold_.

We tested whether *F*_ST>threshold_ SNPs are more clustered than *F*_ST<threshold_ SNPs by comparing the distances between adjacent SNPs of each allele frequency group. Because there are fewer *F*_ST>threshold_ SNPs, we used a random subset of *F*_ST<threshold_ SNPs of equal size. Distances were calculated separately for each allele frequency group and the dispersal of the distance distributions was assessed using three measures: coefficient of variation [56], [57], quartile coefficient of dispersion [58], and geometric coefficient of variation [59]. Measures were calculated for each chromosome, weighted by the proportion of SNPs on that chromosome, and summed over all chromosomes. To estimate the significance of the results, we used a bootstrap approach and repeated the calculation 10,000 times with random subsets of *F*_ST<threshold_ SNPs.

Similarly, we compared the linkage disequilibrium (LD) between adjacent *F*_ST>threshold_ SNPs and *F*_ST<threshold_ SNPs using the squared correlation coefficient (*r^2^*). The LD was calculated separately for each continental population and allele frequency group. We used a bootstrap approach to estimate the significance of the results with 10,000 random subsets of *F*_ST<threshold_ SNPs.

## Supporting Information

Figure S1 **Distribution of genetic variation per HapMap population and phase.** SNPs were classified in ten minor allele groups based on their frequency in each population and further subdivided by HapMap phases: 1 (blue), 2 (green), and 3 (red). The number of SNPs genotyped in each phase (*n_1_.._3_*) and the total number of SNPs (*n_tot_*) are marked.
(TIF)

Figure S2 **Fitting the expected cumulative distribution function of an exponential distribution to the** ***F*** **_ST_ distribution.** The two distributions largely overlap.
(TIF)

Figure S3 ***F*** **_ST_ values of SNPs from the continental dataset versus their expected exponential values.** *F*_ST_ values were calculated for all SNPs (red), excluding rare ones (MAF <0.05) (blue) for autosomal (a) and X-chromosomal (b) SNPs.
(TIF)

Figure S4 **Distribution of locus-specific** ***F*** **_ST_ in eight populations (CEU, CHB, CHD, JPT, LWK, MKK, YRI, and TSI).** *F*_ST_ values were obtained for a. 1,100,484 autosomal SNPs, and b. 32,650 SNPs on the non-recombining region of the X chromosome. The histograms show bin distribution as indicated on the x-axis and the cumulative distribution (line).
(TIF)

Figure S5 **A histogram of the distances between adjacent** ***F*** **_ST>threshold_ SNPs for five allele frequency groups.**
(TIF)

Figure S6 **LD for five allele frequency groups as a function of physical distance in Europeans.** LD (*r^2^*) in European populations is plotted as a function of physical distance on a log-scale for five allele frequency groups (a–e). To simplify the presentation, the mean and standard error of the mean *r^2^* for the *F*_ST >threshold_ SNPs (blue) and *F*_ST<threshold_ (red) are presented for different between-SNP distances (50 bp, 100 bp, 1 kb, 5 kb, 10 kb, 50 kb, 100 kb, 500 kb, and 1000 kb). *F*_ST>threshold_ SNPs are marked as green dots.
(TIF)

Figure S7 **LD for five allele frequency groups as a function of physical distance in Asians.** LD (*r^2^*) in Asian populations is plotted as a function of physical distance on a log-scale for five allele frequency groups (a–e). To simplify the presentation, the mean and standard error of the mean *r^2^* for the *F*_ST >threshold_ SNPs (blue) and *F*_ST<threshold_ (red) are presented for different between-SNP distances (50 bp, 100 bp, 1 kb, 5 kb, 10 kb, 50 kb, 100 kb, 500 kb, and 1000 kb). *F*_ST>threshold_ SNPs are marked as green dots.
(TIF)

Table S1 **Summary of HapMap phase 3 (second draft) data used in our analyses.** The number of SNPs that passed or failed QC++ (top) and the number of unrelated samples that passed or failed QC++ (bottom).
(DOC)

Table S2 **Summary of SNP statistics per chromosome.** Number of SNPs segregating in all samples within the continental dataset, SNPs density, mean and standard deviation of MAF, and mean and standard deviation of *F*_ST_ for each chromosome.
(DOC)

Text S1 **Assessing data quality, F-statistics for measuring population differentiation, and Supporting Information References.**
(DOC)
