# Supplementary figures and images for: Empirical Distributions of F ST from Large-Scale Human Polymorphism Data
Source: PLoS One. 2012 Nov 21;7(11):e49837. doi: 10.1371/journal.pone.0049837 (PMC3504095; doi:10.1371/journal.pone.0049837)

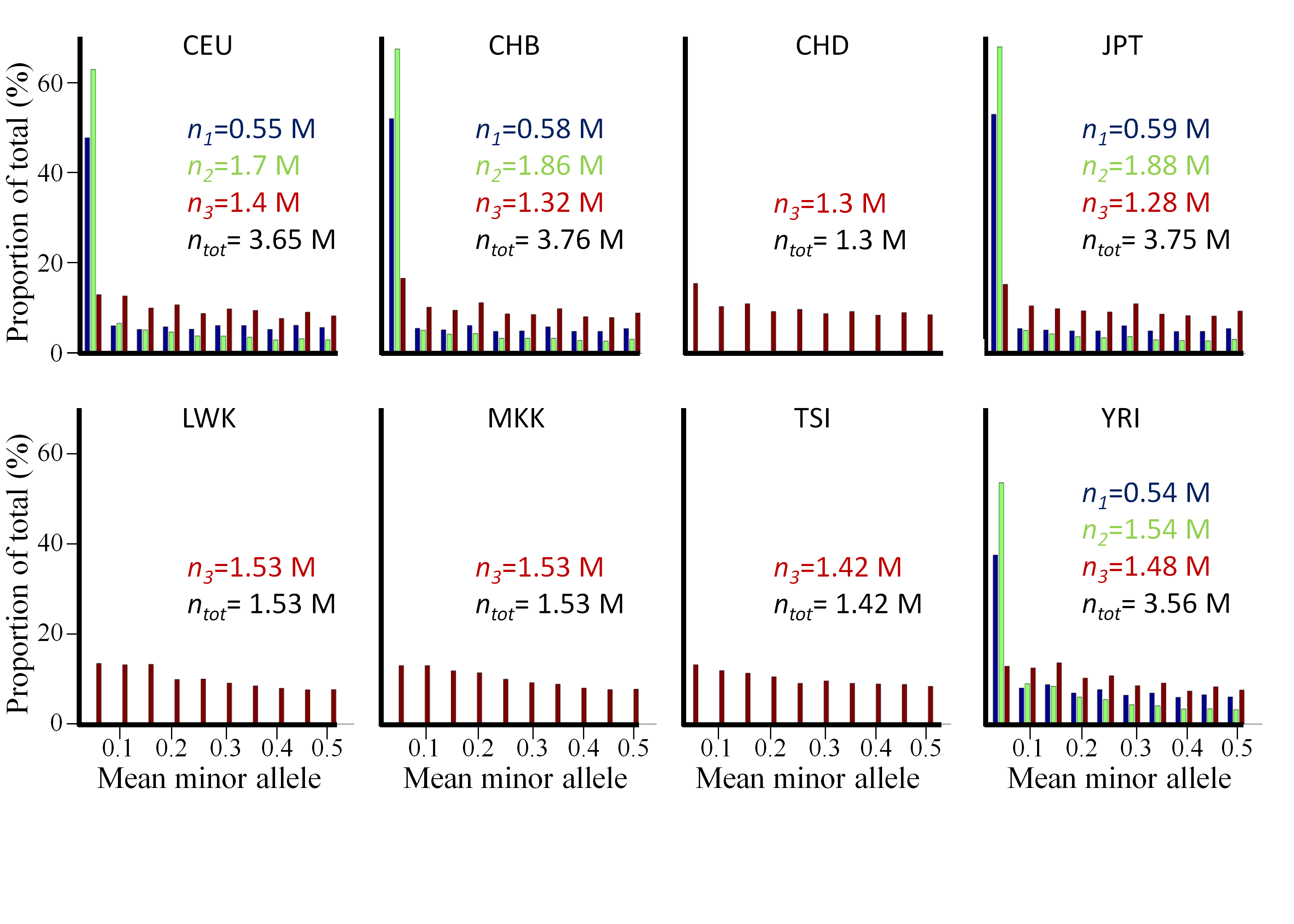

Supplement: Figure S1 — Distribution of genetic variation per HapMap population and phase. SNPs were classified in ten minor allele groups based on their frequency in each population and further subdivided by HapMap phases: 1 (blue), 2 (green), and 3 (red). The number of SNPs genotyped in each phase (n1..3) and the total number of SNPs (ntot) are marked. (TIF) [file pone.0049837.s001.tif]

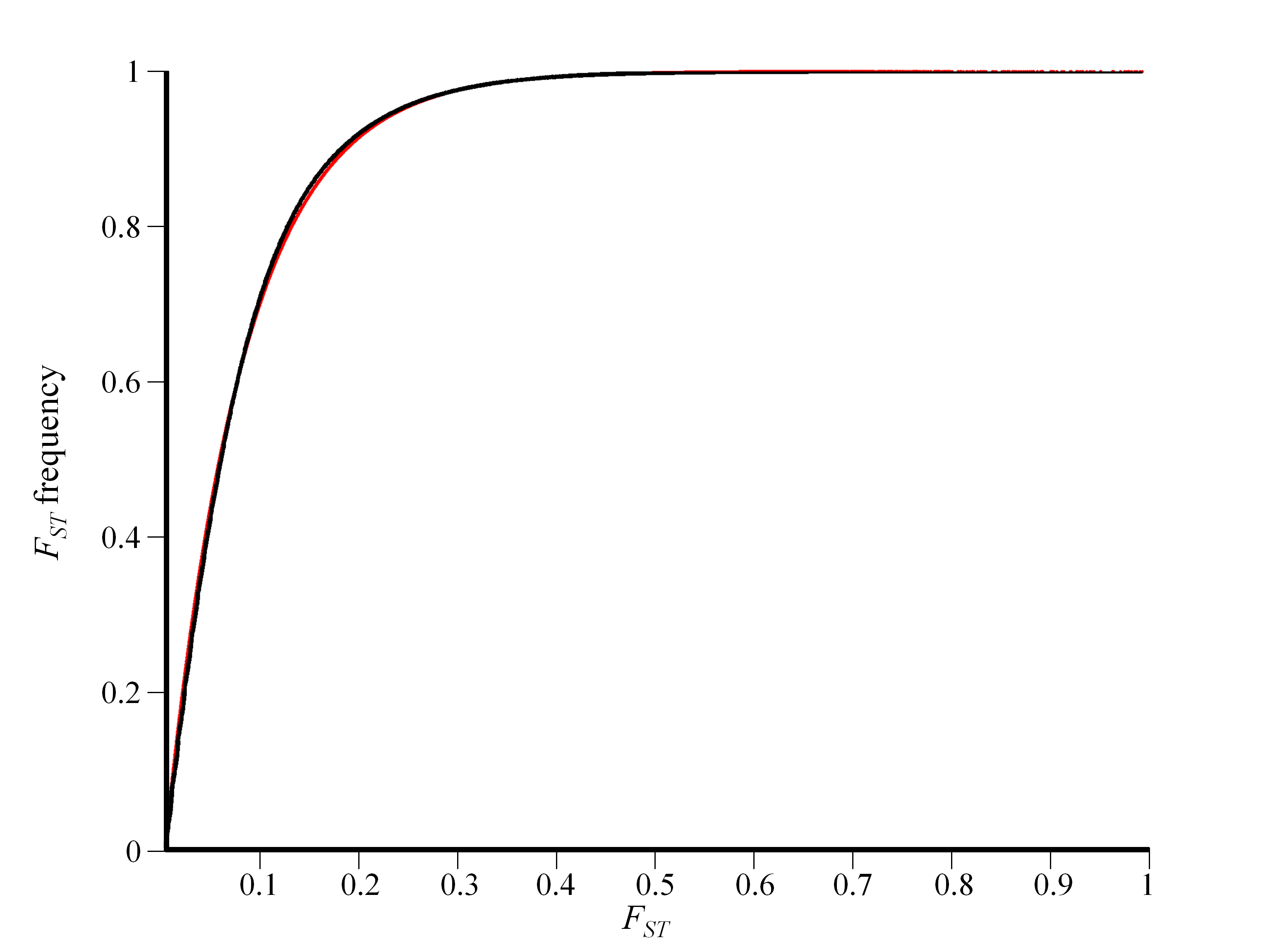

Supplement: Figure S2 — Fitting the expected cumulative distribution function of an exponential distribution to the F ST distribution. The two distributions largely overlap. (TIF) [file pone.0049837.s002.tif]

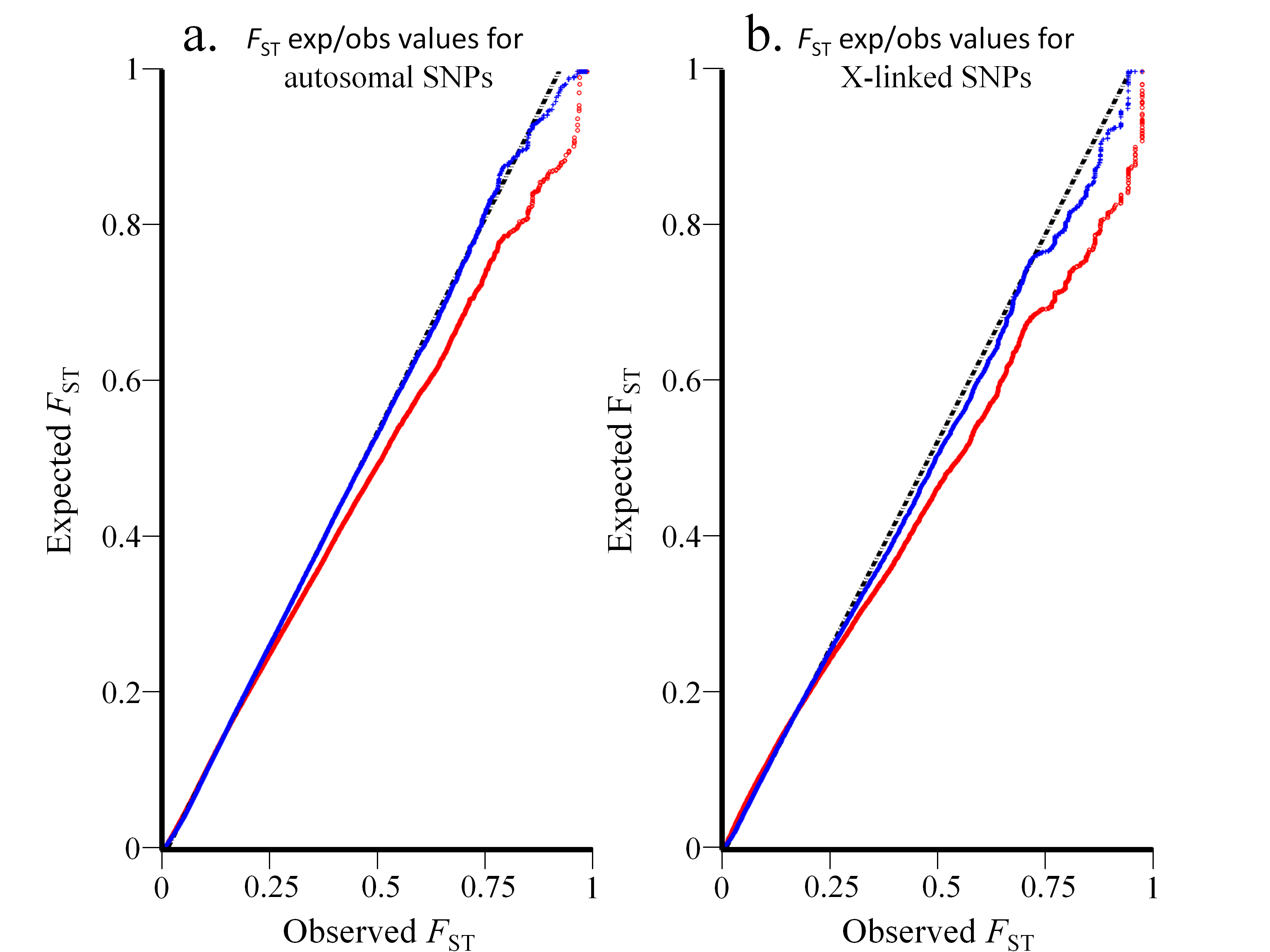

Supplement: Figure S3 — F ST values of SNPs from the continental dataset versus their expected exponential values. F ST values were calculated for all SNPs (red), excluding rare ones (MAF <0.05) (blue) for autosomal (a) and X-chromosomal (b) SNPs. (TIF) [file pone.0049837.s003.tif]

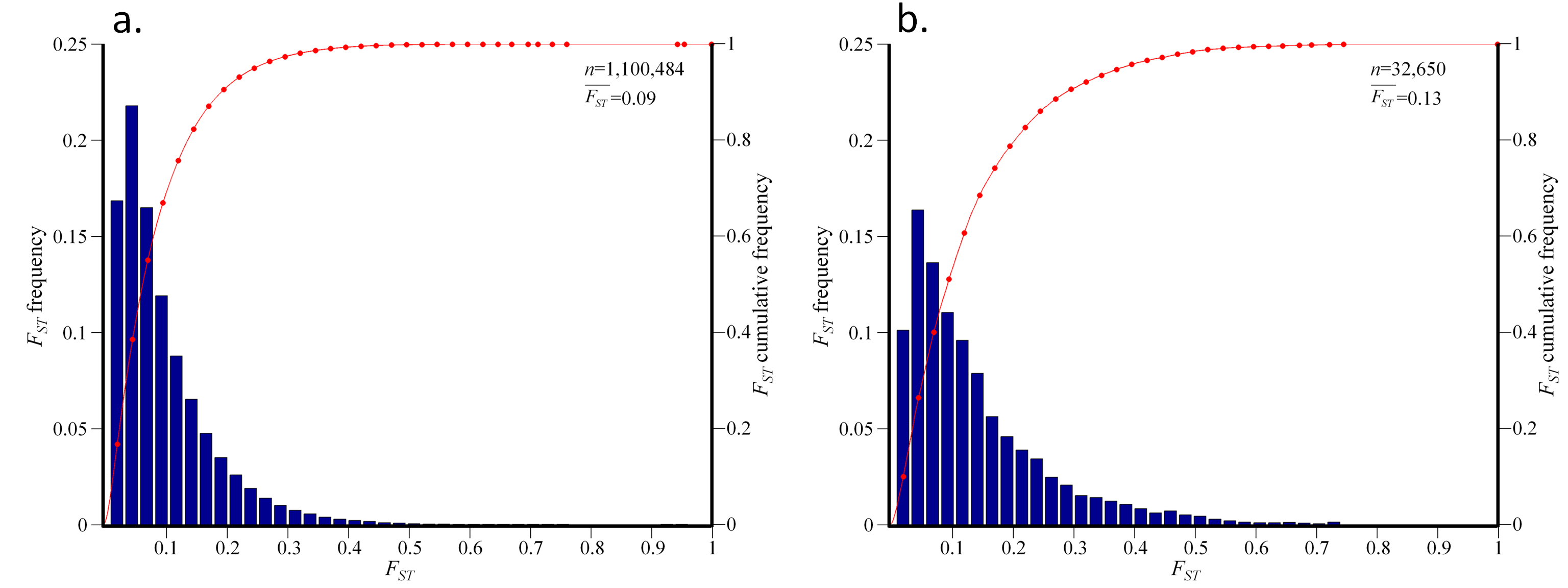

Supplement: Figure S4 — Distribution of locus-specific F ST in eight populations (CEU, CHB, CHD, JPT, LWK, MKK, YRI, and TSI). F ST values were obtained for a. 1,100,484 autosomal SNPs, and b. 32,650 SNPs on the non-recombining region of the X chromosome. The histograms show bin distribution as indicated on the x-axis and the cumulative distribution (line). (TIF) [file pone.0049837.s004.tif]

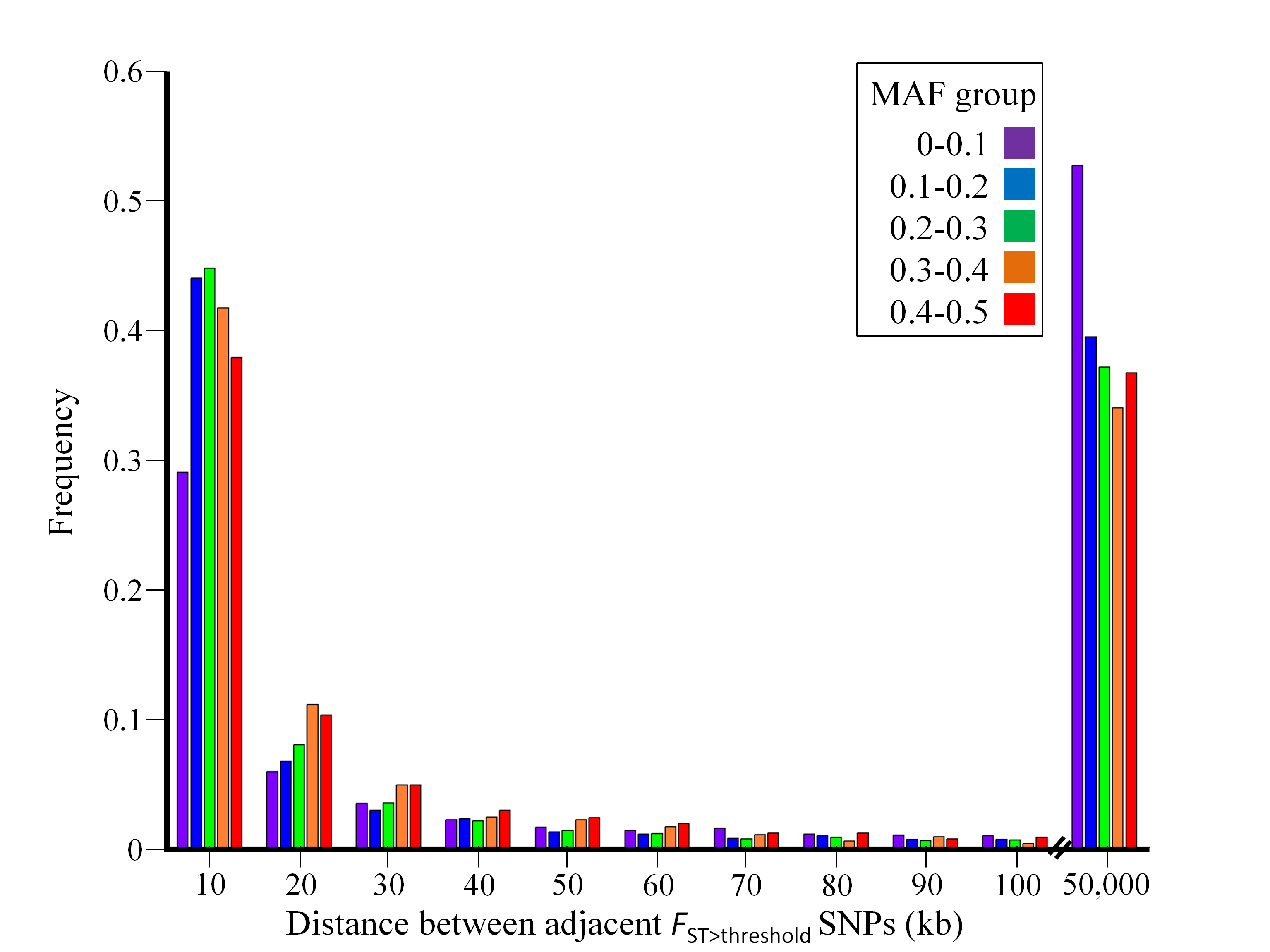

Supplement: Figure S5 — A histogram of the distances between adjacent F ST>threshold SNPs for five allele frequency groups. (TIF) [file pone.0049837.s005.tif]

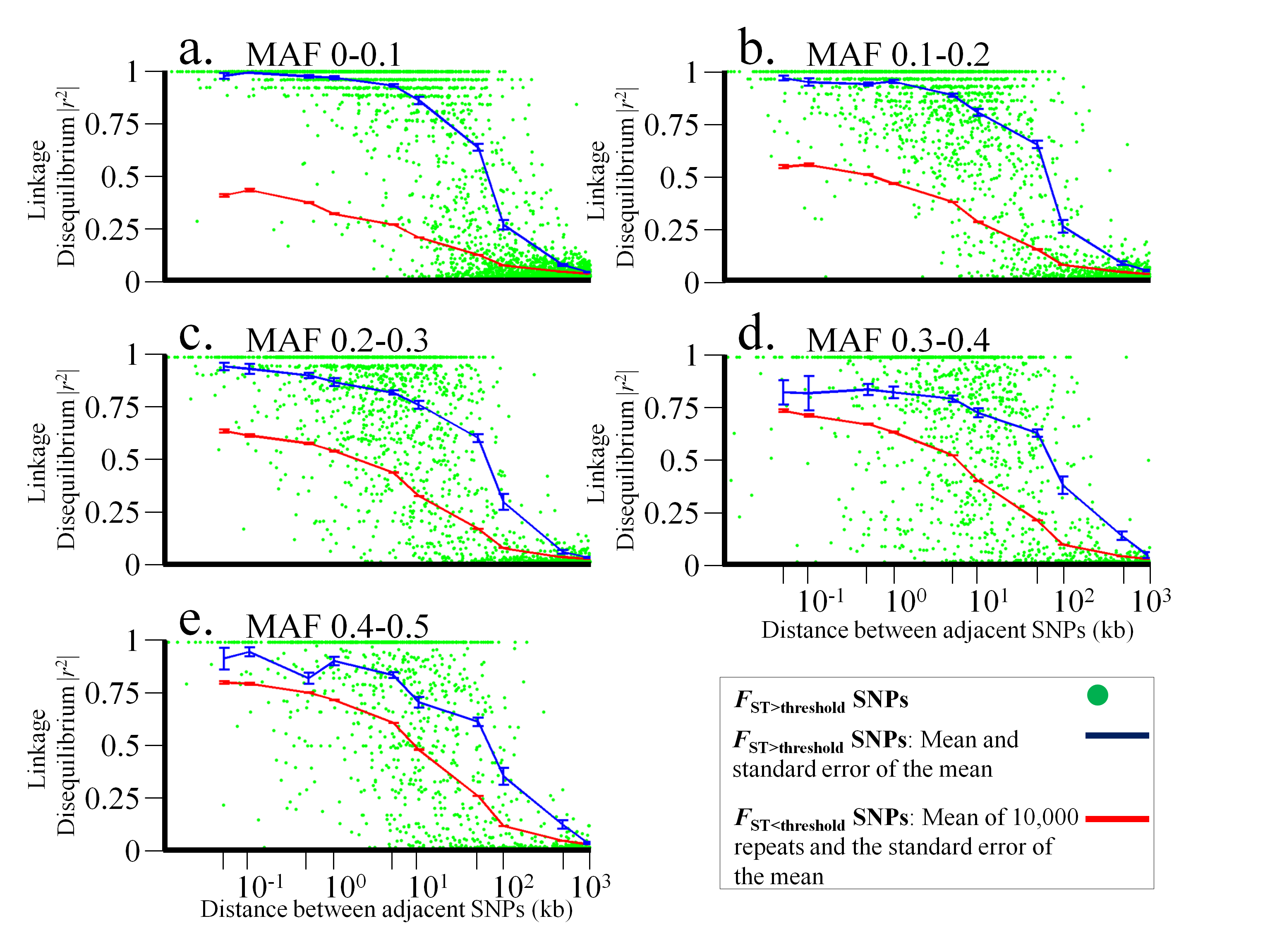

Supplement: Figure S6 — LD for five allele frequency groups as a function of physical distance in Europeans. LD (r2) in European populations is plotted as a function of physical distance on a log-scale for five allele frequency groups (a–e). To simplify the presentation, the mean and standard error of the mean r2 for the F ST >threshold SNPs (blue) and F STthreshold SNPs are marked as green dots. (TIF) [file pone.0049837.s006.tif]

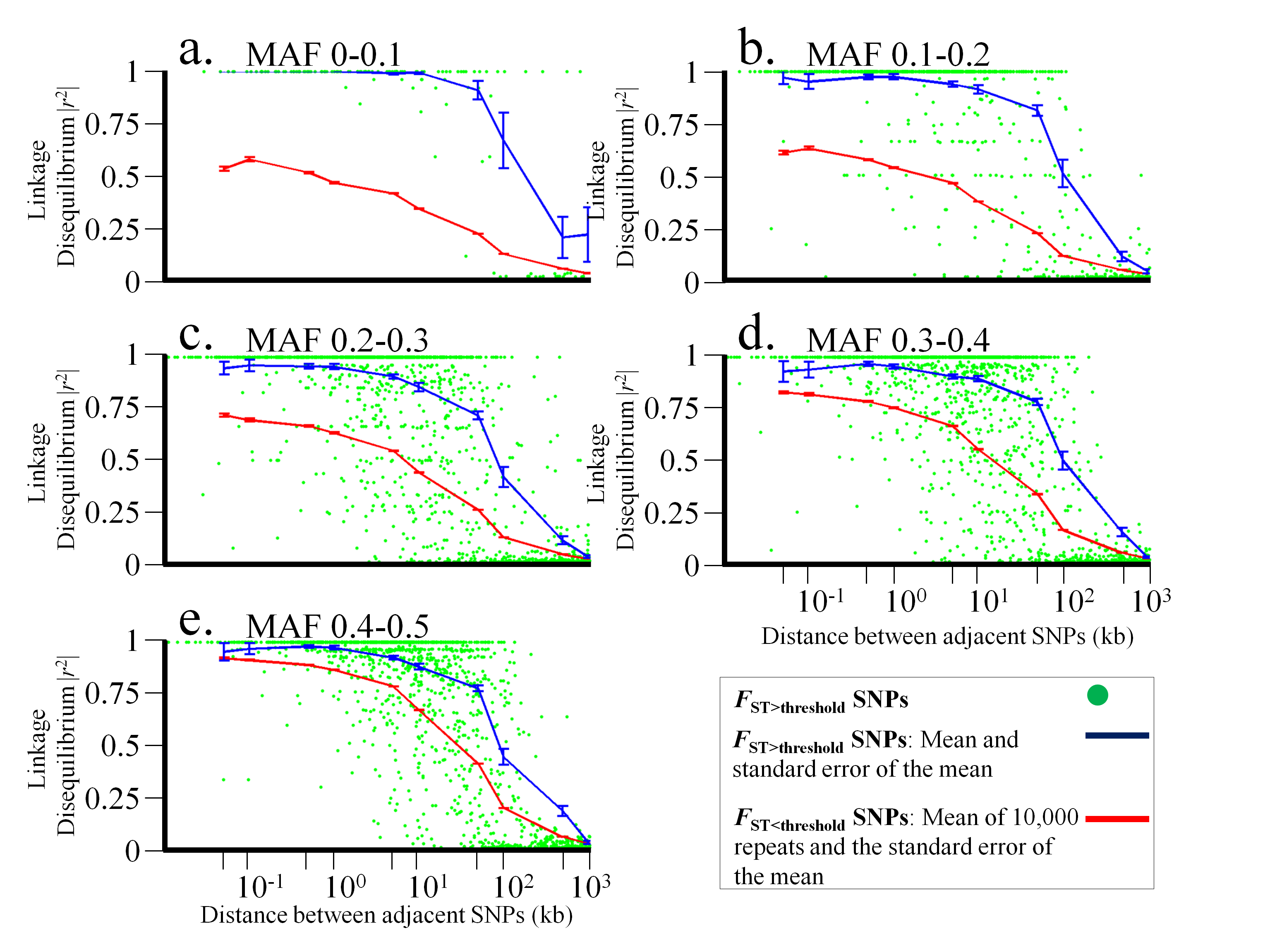

Supplement: Figure S7 — LD for five allele frequency groups as a function of physical distance in Asians. LD (r2) in Asian populations is plotted as a function of physical distance on a log-scale for five allele frequency groups (a–e). To simplify the presentation, the mean and standard error of the mean r2 for the F ST >threshold SNPs (blue) and F STthreshold SNPs are marked as green dots. (TIF) [file pone.0049837.s007.tif]
