# Supplementary material for: Empirical Distributions of F ST from Large-Scale Human Polymorphism Data
Source: PLoS One. 2012 Nov 21;7(11):e49837. doi: 10.1371/journal.pone.0049837 (PMC3504095; doi:10.1371/journal.pone.0049837)
Supplement: Table S1 — Summary of HapMap phase 3 (second draft) data used in our analyses. The number of SNPs that passed or failed QC++ (top) and the number of unrelated samples that passed or failed QC++ (bottom). (DOC) [file pone.0049837.s008.doc]

**Table S1. Summary of HapMap phase 3 (second draft) data used in our analyses**

|  |  |  | Populations | | |  |  |  |
| --- | --- | --- | --- | --- | --- | --- | --- | --- |
|  | CEU | CHB | CHD | JPT | LWK | MKK | TSI | YRI |
| Total SNPs | 4,030,774 | 4,052,336 | 1,306,196 | 4,052,423 | 1,529,764 | 1,537,638 | 1,419,921 | 3,984,356 |
| Passed QC++ (%) | 3,645,280  (90) | 3,759,887 (93) | 1,306,055 (100) | 3,751,746 (93) | 1,529,609 (100) | 1,527,806 (99) | 1,419,778 (100) | 3,554,473 (89) |
| Failed QC++ |  |  |  |  |  |  |  |  |
| >5% missingness (%) | 384,780 (10) | 292,449 (7) | 141 (0) | 300,677 (7) | 155 (0) | 3,532 (1) | 143 (0) | 429,231 (11) |
| >1 Mendel error (%) | 714 (0) |  |  |  |  | 6,300 (0) |  | 652 (0) |
| Total samples | 174 | 86 | 85 | 89 | 90 | 171 | 88 | 176 |
| Passed QC++ (%) | 56 (32) | 43 (50) | 85 (100) | 42 (47) | 90 (100%) | 143 (83) | 88 (100) | 55 (30) |
| Failed QC++ |  |  |  |  |  |  |  |  |
| >5% missingness (%) | 60 (34) | 43 (50) |  | 47 (53) |  |  |  | 63 (36) |
| Related samples (%) | 58 (33) |  |  |  |  | 28 (17) |  | 58 (34) |
