# Supplementary material for: Empirical Distributions of F ST from Large-Scale Human Polymorphism Data
Source: PLoS One. 2012 Nov 21;7(11):e49837. doi: 10.1371/journal.pone.0049837 (PMC3504095; doi:10.1371/journal.pone.0049837)
Supplement: Table S2 — Summary of SNP statistics per chromosome. Number of SNPs segregating in all samples within the continental dataset, SNPs density, mean and standard deviation of MAF, and mean and standard deviation of F ST for each chromosome. (DOC) [file pone.0049837.s009.doc]

**Table S2. Summary of SNP statistics per chromosome**

| Chromosome | No. SNPs | SNP density (kb) | MAF (σ) | *F*_ST_ (σ) |
| --- | --- | --- | --- | --- |
| 1 | 223,552 | 0.904 | 0.21 (0.021) | 0.08 (0.006) |
| 2 | 251,759 | 1.037 | 0.21 (0.022) | 0.08 (0.007) |
| 3 | 188,753 | 0.946 | 0.22 (0.022) | 0.08 (0.006) |
| 4 | 175,729 | 0.919 | 0.21 (0.021) | 0.08 (0.007) |
| 5 | 183,675 | 1.016 | 0.21 (0.022) | 0.08 (0.006) |
| 6 | 199,316 | 1.166 | 0.21 (0.021) | 0.08 (0.006) |
| 7 | 154,636 | 0.974 | 0.21 (0.022) | 0.08 (0.006) |
| 8 | 163,747 | 1.119 | 0.22 (0.022) | 0.08 (0.006) |
| 9 | 135,468 | 0.966 | 0.21 (0.021) | 0.08 (0.006) |
| 10 | 152,239 | 1.125 | 0.2 (0.021) | 0.08 (0.006) |
| 11 | 143,402 | 1.067 | 0.21 (0.022) | 0.08 (0.006) |
| 12 | 134,495 | 1.017 | 0.21 (0.021) | 0.08 (0.006) |
| 13 | 115,307 | 1.01 | 0.2 (0.022) | 0.08 (0.006) |
| 14 | 93,458 | 0.879 | 0.21 (0.022) | 0.08 (0.006) |
| 15 | 80,907 | 0.806 | 0.22 (0.022) | 0.09 (0.007) |
| 16 | 80,547 | 0.907 | 0.21 (0.022) | 0.08 (0.006) |
| 17 | 66,969 | 0.851 | 0.22 (0.022) | 0.08 (0.007) |
| 18 | 87,111 | 1.144 | 0.21 (0.022) | 0.08 (0.006) |
| 19 | 44,027 | 0.69 | 0.22 (0.022) | 0.08 (0.005) |
| 20 | 72,001 | 1.153 | 0.21 (0.022) | 0.08 (0.006) |
| 21 | 37,763 | 0.804 | 0.22 (0.021) | 0.08 (0.006) |
| 22 | 38,506 | 0.777 | 0.21 (0.022) | 0.08 (0.006) |
| X | 86,533 | 0.559 | 0.22 (0.022) | 0.12 (0.018) |
| All | 2,909,900 | 0.96 | 0.21 (0.022) | 0.08 (0.007) |
