# Supplementary material for: Empirical Distributions of F ST from Large-Scale Human Polymorphism Data
Source: PLoS One. 2012 Nov 21;7(11):e49837. doi: 10.1371/journal.pone.0049837 (PMC3504095; doi:10.1371/journal.pone.0049837)
Supplement: Text S1 — Assessing data quality, F-statistics for measuring population differentiation, and Supporting Information References. (DOC) [file pone.0049837.s010.doc]

**Supplementary Text**

*Assessing data quality*

Because we used the second draft of HapMap 3, data quality was achieved by applying two criteria to HapMap’s QC+ SNP data: low missingness rate (< 5%), calculated as the average number of null genotypes over all samples in a population; and less than two Mendelian errors for each SNP in each population, where trio data were available. Two criteria were also applied to sample data: low missingness rate (< 5%), calculated as the average number of null genotypes over all SNPs; and exclusion of offspring. Overall, 3.5-3.8 million SNPs (CEU, CHB, JPT, and YRI) and 1.3-1.5 million SNPs (CHD, LWK, MKK, and TSI) in 602 samples (302 females and 300 males) passed our filtering criteria and were termed “QC++.”

*F-statistics for measuring population differentiation*

Wright [1] proposed three *F*-statistics that quantify the genetic differentiation in a heretical population structure of two levels that are interrelated by the formula:

$1-F_{IT}=(1-F_{IS})(1-F_{ST})$, (S1)

where *F*_IT_ is the inbreeding coefficient of individuals compared to the total population ranging from –1 (individuals are heterozygous) and 1 (individuals are homozygous), *F*_IS_ is the inbreeding coefficient of individuals relative to a subpopulation ranging from –1 (homozygote deficiency) and 1 (heterozygote deficiency), and *F*_ST_ quantifies the differentiation between subpopulations in the total population ranging from 0 (subpopulations have the same allele frequencies) to 1 (subpopulations have different allele frequencies). With the absence of migration between populations and similar initial allele frequency in all populations, *F*_ST_ after *t* generations is given by

$1-F_{\text{ST}}=(1-\frac{1}{2N_{e}})^{t}\approx e^{-t/2N_{e}}$, (S2)

where *N*_e_ is the effective population size.

To estimate the genetic differentiation in three hierarchical levels, we defined six fixation indices: *F*_IS_ - individuals relative to intra-continental populations, *F*_SC_ - intra-continental populations relative to the continental population, *F*_CT_ - continental population relative to the total population, $F_{IT}=1-(1-F_{IS})(1-F_{ST})$- individuals relative to the total population, $F_{IC}=1-(1-F_{IS})(1-F_{SC})$individuals relative to continental population, and $F_{ST}=1-(1-F_{SC})(1-F_{CT})$intra-continental populations relative to the total population. The relationships between the indices derive from Eq. S1 and are also described in Figure 1. The two indices that measure the nested contribution to genetic differentiation at the different geographical levels are *F*_SC_ and *F*_CT_.

**Supplementary Information References**

1. Wright S (1965) The interpretation of population structure by F-statistics with special regard to systems of mating. Evolution 19: 395-420.
